# Ileoileal knotting: a rare cause of intestinal obstruction: a case report

**DOI:** 10.1186/s13256-021-02910-6

**Published:** 2021-07-22

**Authors:** Yusuf Mohammed, Kirubel Tesfaye

**Affiliations:** Department of Surgery, Ethio-Tebib General Hospital, Addis Ababa, Ethiopia

**Keywords:** Small bowel obstruction, Ileoileal knotting, Gangrenous bowel

## Abstract

**Background:**

Ileoileal knotting is one of the rarest causes of intestinal obstruction. The pathology involves knotting of the ileum around itself, leading to mechanical intestinal obstruction that can rapidly evolve to gangrene.

**Case presentation:**

Here we will discuss the case of an 18-year-old Oromo girl who presented with sudden onset of severe abdominal pain and signs of generalized peritonitis.Ultrasound examination showed massive peritoneal and cul-de-sac fluid. Explorative laparotomy was done, with a tentative diagnosis of ruptured ovarian cyst. Intraoperative finding was a gangrenous ileoileal knot. The gangrenous segment was resected and ileotransverse anastomosis done. Postoperative course was uneventful, and the patient was discharged improved on the sixth postoperative day.

**Conclusion:**

We present this case to highlight the diagnostic difficulty that one can face in females of child-bearing age and to create awareness of this rare cause of intestinal obstruction, as morbidity and mortality are very high because of rapid progression to gangrene.

## Background

One of the most common surgical emergencies worldwide is intestinal obstruction, with small bowel obstruction (SBO) accounting for the majority of cases [1]. Intestinal knotting is a very rare cause. The different types of knotting that can be discovered during laparotomy are ileosigmoid, ileoileal, and cecosigmoid [2]. Ileoileal type is the rarest; only one such case has been reported in Ethiopia [2] and a few more worldwide. Here we will discuss the unique case of an 18-year-old female with strangulated intestinal obstruction due to ileoileal knotting that required surgical intervention.

## Case presentation

**Fig. 1:**
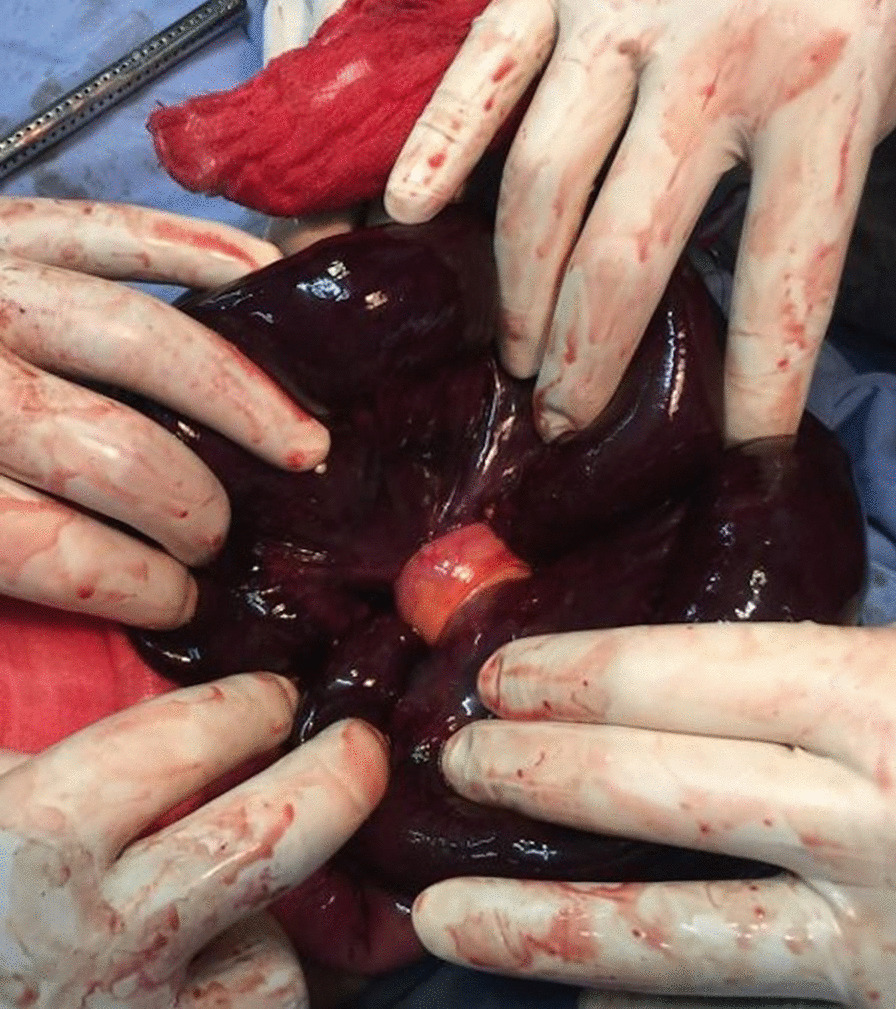
Ileoileal knot and gangrenous bowel

An 18-year-old Oromo high school student initially presented to the emergency gynecology department of Ethio-Tebib Hospital, Addis Ababa, Ethiopia with 12 hours history of severe crampy abdominal pain. It was initially periumbilical and later became generalized. Associated with this, she had repeated vomiting that initially consisted of ingested matter and later became bilious. She also had two episodes of watery diarrhea, yellowish in color. She had history of irregular menses, and her last cycle was 6 months back. She did not have history of previous abdominal surgery and gave no history of abdominal trauma. On physical examination., blood pressure (BP) was 120/70 mmHg, heart rate 112/minute, respiratory rate (RR) 26 breaths/minute, temperature 36.5 °C ,and oxygen saturation 98%. She had pink conjunctivae and dry buccal mucosa. Pertinent physical examination finding was on the abdomen, which was full and showed no movement with respiration, with diffuse direct and rebound tenderness. Bowel sounds were hypoactive, and digital rectal examination was tender anteriorly and showed no mass with well-formed stool on examining finger. Per vaginal examination was deferred as she was a virgin. Laboratory tests were all normal, including urine human chorionic gonadotropin (HcG) which was negative; however, abdominopelvic ultrasound showed massive free peritoneal and cul-de-sac fluid. Diagnostic paracentesis showed hemorrhagic fluid. With a diagnosis of acute abdomen with generalized peritonitis and hemoperitoneum secondary to ruptured ovarian cyst, she was resuscitated with intravenous normal saline and taken to the operating theater. Upon exploration, there was 500 ml of hemorrhagic fluid and gangrenous distal ileum seen caught in a knot formed by the proximal ileum (Fig. 1).

**Fig. 2:**
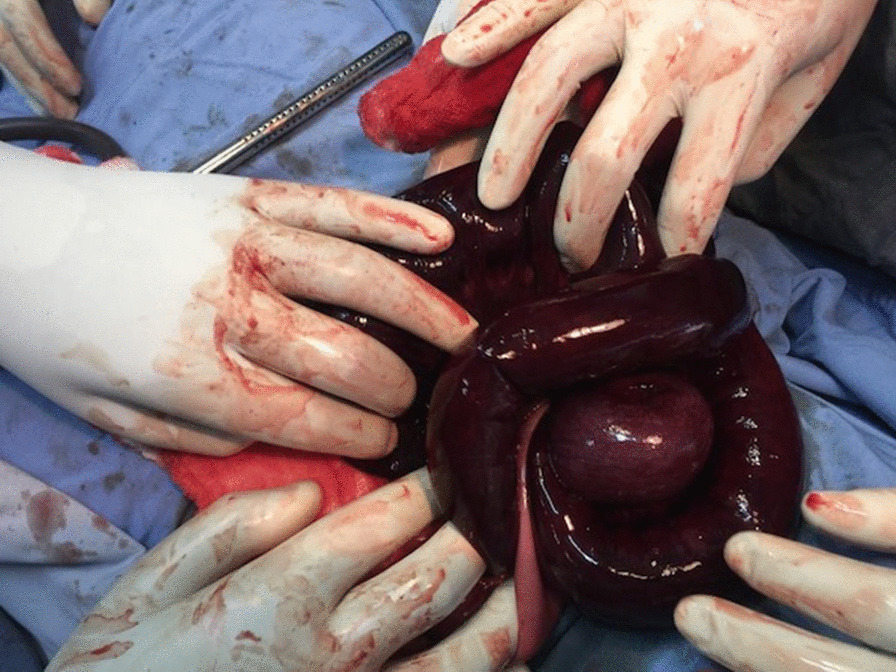
After the ileoileal knot was released

Intraoperative consultation of surgical department was done. The entangled segment was released and around 100 cm of gangrenous bowel resected (Fig. 2). There was around 3 cm of viable distal ileum, and thus, right hemicolectomy and ileotransverse anastomosis were done in two layers. Postoperatively, she was started on fluid diet on the third day and solid food on the sixth and discharged home after full recovery. Follow-up appointments at third and sixth month were uneventful.

## Discussion

Intestinal obstruction remains one of the leading surgical emergencies worldwide, and the subset small bowel obstruction is the most commonly encountered surgical disorder of the small intestine [3]. Studies have shown that up to 80% of intestinal obstructions are due to SBO [1]. In Ethiopia, SBO remains the most common type, and the leading causes are adhesions, small bowel volvulus, and hernia [4]. To our knowledge, there is only one reported case of ileoileal knotting in Ethiopia [2], and only a few cases have been reported worldwide. One study published in 2017 reported of only seven cases worldwide [5]. The citing of ileoileal knotting was first reported in the sixteenth century by Riverius, and by Rokintasky in 1836 [6]. Different types of knotting are described: ileoileal, ileosigmoid, cecosigmoid, and a loop formed between bowel and Meckel’s diverticulum or appendix [2]. Ileoileal knotting is the rarest of the intestinal knotting [3].

Diagnosis of ileoileal knotting is done intraoperatively. The exact cause remains unknown, but there are few stated hypotheses. Among them is the type of diet the patient consumes, that is, high fiber, which could also be the case in our setup [7]. The morbidity and mortality of ileoileal knotting and intestinal obstruction as a whole are quite high especially, when they go undiagnosed or diagnosis is delayed because of low index of suspicion. Blood supply being cut off to the obstructed segment subsequently leads to gangrene and generalized peritonitis. Delay in diagnosis will increase the morbidity and mortality. A local study that evaluated the management of small bowel obstruction reported mortality of up to 9.5%, and 34.9% of patients had complications at presentation [4]. Also noted was that around 50% of the study population presented after 24 hours of their illness [4]. Other studies show mortality ranging from 8% to 25% [3].

After intraoperative diagnosis, management is based on the viability of the bowel. When the bowel loops are viable, careful untying of the loops is performed as there is very low risk of recurrence. In the case of gangrenous changes, standard of treatment is controlled enterotomy decompression followed by resection and primary anastomosis guided by the length of ileum left after resection [8]. In situations where the lesion is less than 10–15 cm away from the ileocecal valve, standard of treatment is right hemicolectomy and ileotransverse anastomosis [9] as was done in our patient. Attempts of ileoileal anastomosis in such cases have a risk of postoperative leak as the terminal ileum has poor vascularization. The other possibility is ileoileostomy [9, 10].

Presentation of ileoileal knotting is not significantly different from high intestinal obstructions, but the high morbidity and mortality as a result of rapid progression into gangrene warrant a high index of suspicion. The diagnostic difficulty noticed in our case was due to the presentation overlap with gynecologic emergencies, especially in a woman of reproductive age. Since our patient presented with abdominal cramping and vomiting, factors such as sex of the patient, ultrasound showing fluid in the peritoneal cavity and cul-de-sac, and diagnostic paracentesis revealing blood should not have ruled out the diagnosis of intestinal obstruction before exploration. This indicates the need for a broader perspective, particularly in such patients.

## Conclusion

This second report of ileoileal knotting in Ethiopia should serve as a reminder of the possibility of the diagnosis, and to highlight the importance of early diagnosis and timely intervention to prevent unacceptable morbidity and mortality associated with this disease.

## Data Availability

Not applicable.

## Abbreviation

SBO: Small bowel obstruction

## References

[1] Ullah S, Khan M. Intestinal obstruction: a spectrum of causes. JPMI. 2008;8(1):210–3.

[2] Abebe E, Asmare B, Addise A. Ileo-ileal knotting as an uncommon cause of acute intestinal obstruction. J Surg Case Rep. 2015;2015(8):1. 10.1093/jscr/rjv102 PMC4526845 26251466

[3] Schwartz SI, Brunicardi FC. Schwartz’s principles of surgery. 9th ed. New York: McGraw-Hill Medical; 2011.

[4] Yilma Y. Management outcome of small intestinal obstruction in Mizan Aman General Hospital, Ethiopia. J Clin Exp Pathol. 2018;8(6):359.

[5] Taniguchi K, Lida R, Watanabe T, Nitta M, Tomioka M, Uchiyama K, *et al*. Ileo-ileal knot: a rare case of acute strangulated intestinal obstruction. Nagoya J Med Sci. 2017;79(1):109–13. 10.18999/nagjms.79.1.109 PMC5346628 28303069

[6] Kalaichelvan L, Perumal S, Ross K, Subramanian B. Ileo-ileal knot causing intestinal obstruction a case report. IOSR-JDMS. 2016;15(7):26–9.

[7] Yeo CJ. Shackelfords surgery of the alimentary tract. 7th ed. Philadelphia: Elsevier Saunders; 2012. p. 875.

[8] Nallagounder E. A rare case of ileal gangrene due to ileo-ileal knotting. University J Surg Surg Spec. 2019;5:124–8.

[9] Wei-Wei JW, Xu XQ, Geng QM, Zhang J, Chen H, Lv XF, *et al*. Enteroenteroanastomosis near adjacent ileocecal valve in infants. World J Gastroenterol. 2012;18(48):7314–8. 10.3748/wjg.v18.i48.7314 PMC3544036 23326139

[10] Zhong SZ. Clinical anatomy. Beijing: People’s Medical Publishing House; 1998. p. 366–7.

